# Supplementary material for: Systematic Investigation of the Effects of Multiple SV40 Nuclear Localization Signal Fusion on the Genome Editing Activity of Purified SpCas9
Source: Bioengineering (Basel). 2022 Feb 21;9(2):83. doi: 10.3390/bioengineering9020083 (PMC8869684; doi:10.3390/bioengineering9020083)
Supplement: Supplementary file 1 [file bioengineering-09-00083-s001.zip › bioengineering-1465522-supplementary.pdf]

bioengineering MDPI

Supplementary information

Systematic Investigation of the Effects of Multiple SV40 Nuclear Localization Signal Fusion on the Genome Editing Activity of Purified SpCas9

Sailan Shui[1, 2, 3], Shao-jie Wang[1, 2], Jia Liu[1,*]

[1]Shanghai Institute for Advanced Immunochemical Studies and School of Life Science and Technology, ShanghaiTech University, Shanghai, 201210, China

[2]Contributed equally to this work

[3]Current address: Laboratory of Protein Design and Immunoengineering (LPDI) - STI - EPFL, Lausanne, Switzerland; Swiss Institute of Bioinformatics (SIB), Lausanne CH-1015, Switzerland

*Correspondence should be addressed to J.L. (liujia@shanghaitech.edu.cn)

Supplemented Figures

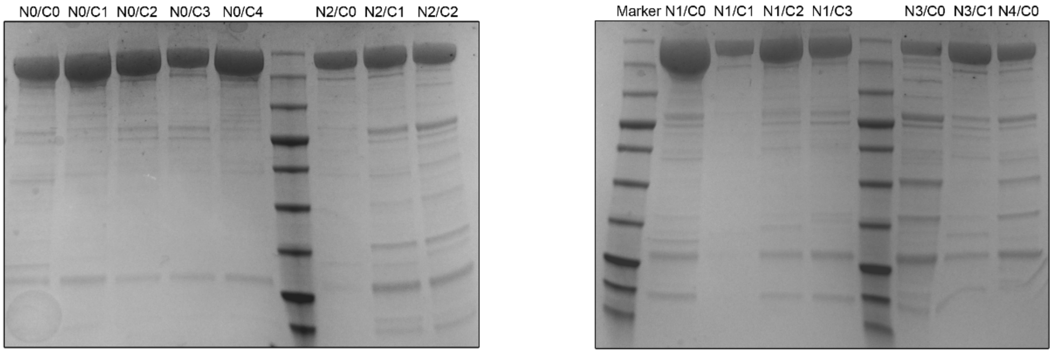

Figure S1. SDS-PAGE of purified SpCas9 proteins in this study.

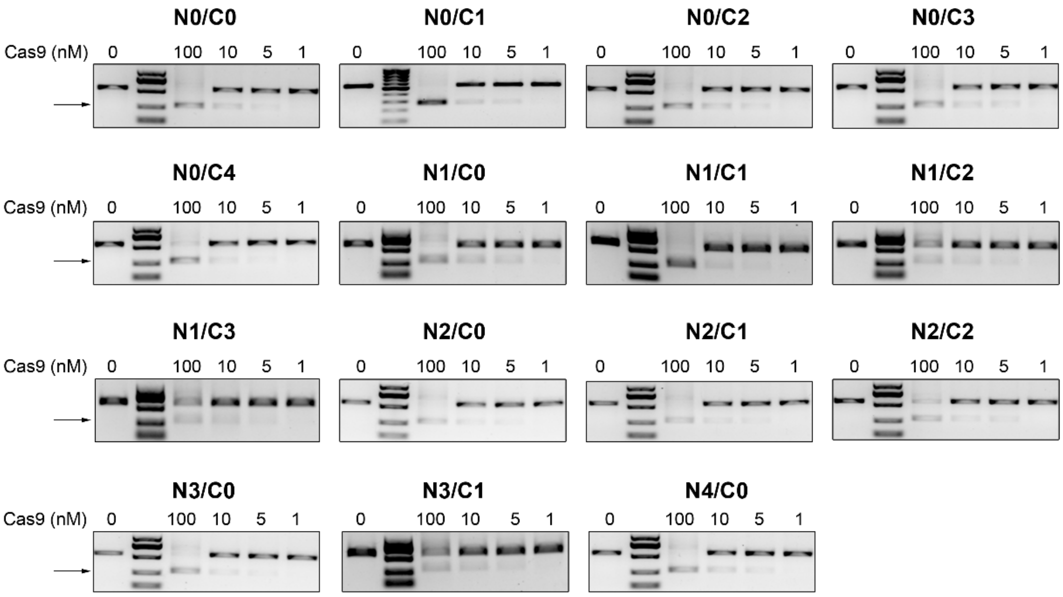

Figure S2. Evaluation of the *in vitro* activity of multi-NLS SpCas9 variants, determined by 1% agarose gel electrophoresis. Cleaved substrate DNA are indicated by arrows.

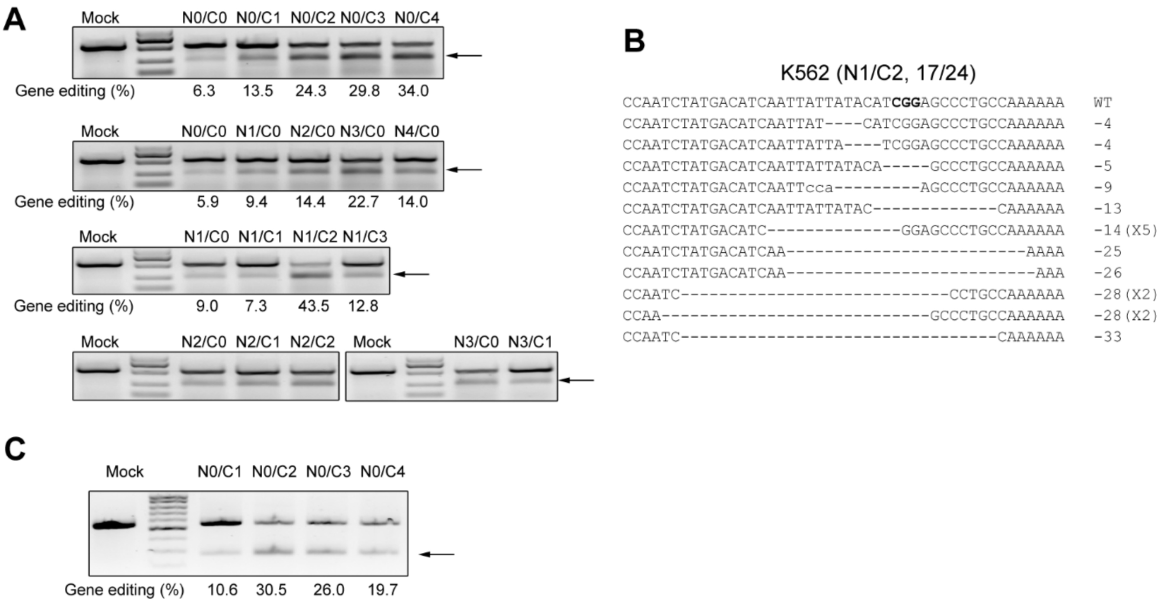

Figure S3. Evaluation of the genome-editing activity of nucleofected multi-NLS SpCas9 proteins in K562 (A-B) and Jurkat (C) cells. (A) Representative images of T7E1 analysis of the genome-editing activity of SpCas9 variants in K562 (Related to Figure 3A). The cleavage product is indicated by arrows. (B) Validation of the modified genomic locus by Sanger sequencing (Related to Figure 3A). (C) Representative images of T7E1 analysis of the genome-editing activity of SpCas9 variants in Jurkat (Related to Figure 3B). The cleavage product is indicated by arrows. T7E1 assay is performed with 1% agarose gel electrophoresis unless noted otherwise.

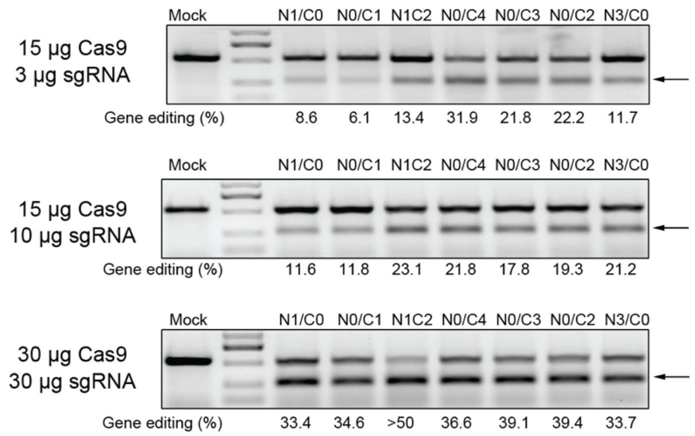

Figure S4 (related to Figure 4A). The effects of multi-NLS on SpCas9 proteins at different dosage, determined by T7E1 assay on 1% agarose gel.

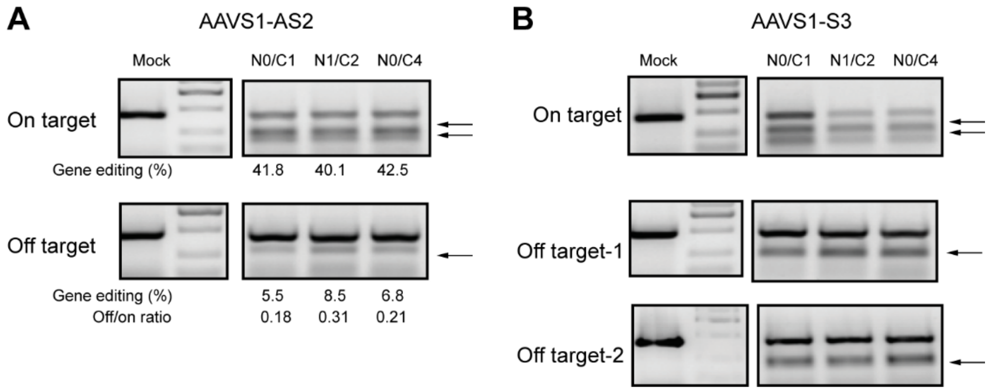

Figure S5 (related to Figure 4B). The effects of multi-NLS on the specificity of SpCas9 variants, determined by T7E1 assay on 1% agarose gel. (A) On-target and off-target activities of sgRNA AAVS1-AS2. (B) On-target and off-target activities of sgRNA AAVS1-S3. The arrows indicate cleavage products.

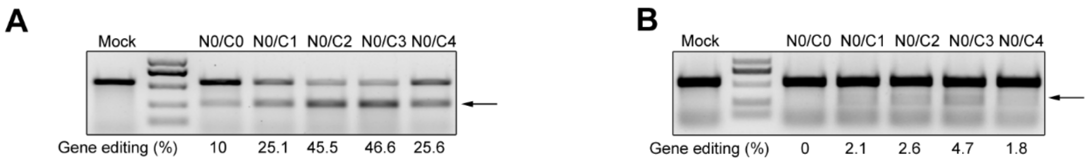

Figure S6 (related to Figure 5). Representative images of the T7E1 results of gene-modified hNPCs (A) and hDFs (B) with multi-NLS SpCas9 proteins. Arrows indicate cleavage product. T7E1 results are resolved on 1% agarose gel.

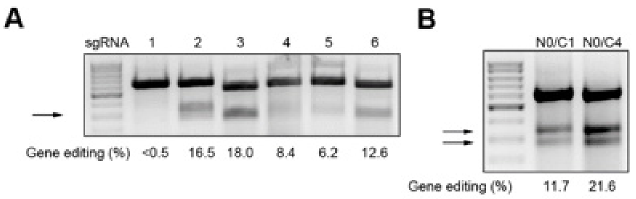

Figure S7 (related to Figure 6). Genome editing of mouse embryos using microinjected N0/C1 and N0/C4 SpCas9 proteins. (A) T7E1 analyses of PTEN-targeting sgRNA screening in mouse Hepa 1-6 cell line. (B) T7E1 analyses of pooled 2-cell embryos treated with N0/C1 and N0/C4 SpCas9 proteins respectively. Arrows indicate cleavage product. T7E1 results are resolved on 1% agarose gel.

Uncropped western blot (related to Figure 3C-D)

Figure 3C

HA   Lamin B1

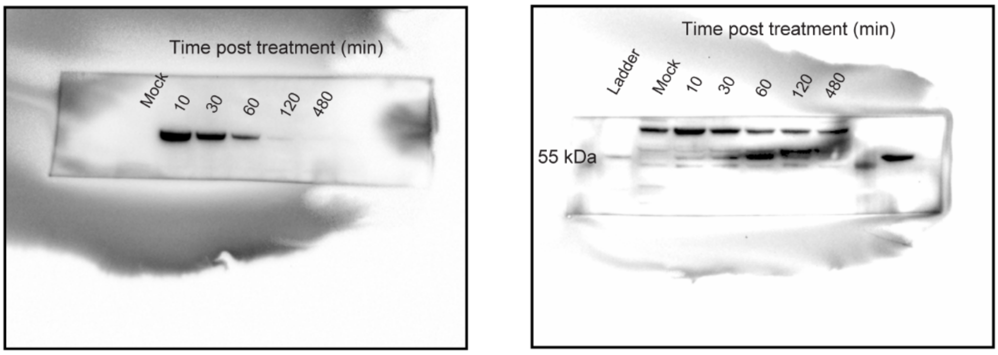

Figure 3D

HA   Lamin B1

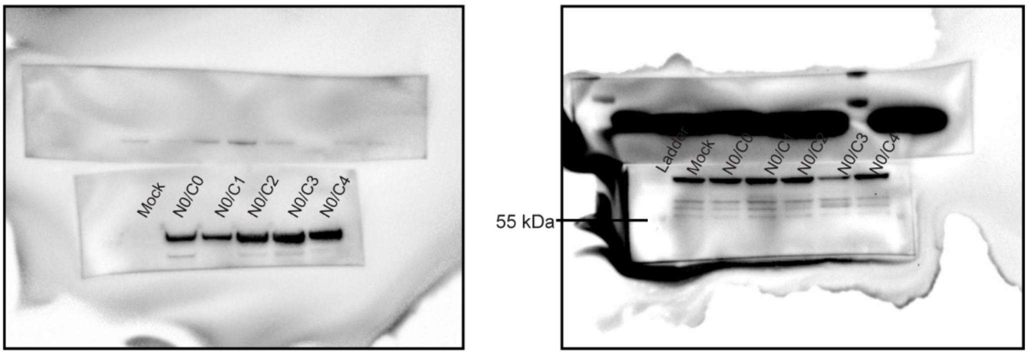

Supplementary Tables

Table S1. sgRNA used in this study

Name	Target gene	Sequence
CCR5-sgRNA	CCR5	TGACATCAATTATTATACAT
293-Reporter-sgRNA-1	EGFP	CAGAATTGATACTGACTGTA
293-Reporter-sgRNA-2	EGFP	GATTCAGTCAGTATCAATTC
AAVS1-AS2	AAVS1	CTCCCTCCCAGGATCCTCTC
AAVS1-S3	AAVS1	GGGAGGGAGAGCTTGGCAGG
EMX1-sgRNA	EMX1	GTCACCTCCAATGACTAGGG
mouse-pTen-sgRNA-1	PTEN	AATATTATTGCTATGGGATT
mouse-pTen-sgRNA-2	PTEN	ACAATATTGATGATGTAGTA
mouse-pTen-sgRNA-3	PTEN	ACCGCCAAATTTAACTGCAG
mouse-pTen-sgRNA-4	PTEN	GCAGCAATTCACTGTAAAGC
mouse-pTen-sgRNA-5	PTEN	TGTCATCTTCACTTAGCCAT
mouse-pTen-sgRNA-6	PTEN	CATACCTCTGCAGTTAAATT

Table S2. Primers used in this study

Name	Sequence
CCR5-EX-F	AGGTGAGAGGATTGCTTG
CCR5-EX-R	AAATGAGAGCTGCAGGTG
CCR5-IN-F	GAGCCAAGCTCTCCATCTAGT
CCR5-IN-R	GCCCTGTCAAGAGTTGACAC
AAVS1-EX-F	AGCCTGAGCGCCTCTCCT
AAVS1-EX-R	TTGCTTTCTTTGCCTGGA
AAVS1-IN-F	TGCTTCTCCTCTTGGGAAGT
AAVS1-IN-R	CGGTTAATGTGGCTCTGGTT
EMX1-EX-F	AGAGGAGCTAGGATGCAC
EMX1-EX-R	TGAATTACCCTTGACCCC
EMX1-IN-F	GGAGCAGCTGGTCAGAGGGG
EMX1-IN-R	AATCTACCACCCCAGGCTCT
AAVS1-AS2-OT-EX-F	CAGGCTCCTTGTTCTTCTGG
AAVS1-AS2-OT-EX-R	AAGCCATGGTCTGCTTCACT
AAVS1-AS2-OT-IN-F	TGCAGGAAAAAGTTGCAGTG
AAVS1-AS2-OT-IN-R	GGCCAAACTGGAGAGACAGA
AAVS1-S3-OT1-EX-F	CAGCTTCCTGGAAAGACCTG
AAVS1-S3-OT1-EX-R	GGTTTTGGCCTGACTGTGTT
AAVS1-S3-OT1-IN-F	TATCTCCTCTCCCCCTGCTT
AAVS1-S3-OT1-IN-R	GAGCTGCCTGACTTCCCTAA
AAVS1-S3-OT2-EX-F	AGCTTCTGCAACTGGTGGTT
AAVS1-S3-OT2-EX-R	AGTTGCTGGTAGGAGGCTGA
AAVS1-S3-OT2-IN-F	GGATGAGAGAGCTGGTTTGC
AAVS1-S3-OT2-IN-R	AGGCTTCTCTTCCCTCGTTC
EMX1-EX-F	TTGAGATGGCTGTTCAGAGG
EMX1-EX-R	TTGAGACATGGGGATAGAATCA
EMX1-IN-F	GTGGGGAGATTTGCATCTGT
EMX1-IN-R	CAATGGGAAGGACAGCTTCT
pTen-1/2-EX-F	CCAGAATATGTAGGCGGAGG
pTen-1/2-EX-R	GTGGGCCTTGAGAAACACTT
pTen-1/2-IN-F	GAGCCTCAGTCGCGTATTCT
pTen-1/2-IN-R	CAAGCCAGAGAATAACCTGG
pTen-3/6-EX-F	AACATGTATGTTTTAAGTAA
pTen-3/6-EX-R	TGTACATTTAAAGCTAAGTG
pTen-3/6-IN-F	GCATGCATGCGTTTAACTTT
pTen-3/6-IN-R	AGCGCTAGCTGTCCCACACC
pTen-4/5-EX-F	CAAGTCACTGGGTAATTTTA
pTen-4/5-EX-R	CATGCCACTAATTCAACAGT
pTen-4/5-IN-F	CCATGTCTAATGAATGCATT
pTen-4/5-IN-R	CAGTTCTCAAAGCATCACAC

Mycoplasma test-F	GGCGAATGGGTGAGTAACACG
Mycoplasma test-R	CGGATAACGCTTGCGACCTATG

Supplementary Methods

1. Sequences of Cas9 proteins

SpyCas9 core sequence

MDKKYSIGLDIGTNSVGWAVITDEYKVPSKKFKVLGNTDRHSIKKNLIGALLFDSGETAEATRLKR
TARRRYTRRKNRICYLQEIFSNEMAKVDDSFFHRLEESFLVEEDKKHERHPIFGNIVDEVAYHEKYP
TIYHLRKKLVDSTDKADLRLIYLALAHMIKFRGHFLIEGDLNPDNSDVDKLFIQLVQTYNQLFEENPI
NASGVDAKAILSARLSKSRRLENLIAQLPGEKKNGLFGNLIALSLGLTPNFKSNFDLAEDAKLQLSK
DTYDDDLDNLLAQIGDQYADLFLAAKNLSDAILLSDILRVNTEITKAPLSASMIKRYDEHHQDLTLL
KALVRQQLPEKYKEIFFDQSKNGYAGYIDGGASQEEFYKFIKPILEKMDGTEELLVKLNREDLLRK
QRTFDNGSIPHQIHLGELHAILRRQEDFYPFLKDNREKIEKILTFRIPYYVGPLARGNSRFAWMTRKS
EETITPWNFEEVVDKGASAQSFIERMTNFDKNLPNEKVLPKHSLLYEYFTVYNELTKVKYVTEGMR
KPAFLSGEQKKAIVDLLFKTNRKVTVKQLKEDYFKKIECFDSVEISGVEDRFNASLGTYHDLLKIIK
DKDFLDNEENEDILEDIVLTLTLFEDREMIEERLKTYAHLFDDKVMKQLKRRRYTGWGRLSRKLIN
GIRDKQSGKTILDFLKSDGFANRNFMQLIHDDSLTFKEDIQKAQVSGQGDSLHEHIANLAGSPAIKK
GILQTVKVVDELVKVMGRHKPENIVIEMARENQTTQKGQKNSRERMKRIEEGIKELGSQILKEHPV
ENTQLQNEKLYLYYLQNGRDMYVDQELDINRLSDYDVDHIVPQSFLKDDSIDNKVLTRSDKNRGK
SDNVPSEEVVKKMKNYWRQLLNAKLITQRKFDNLTKAERGGLSELDKAGFIKRQLVETRQITKHV
AQILDSRMNTKYDENDKLIREVKVITLKSKLVSDFRKDFQFYKVREINNYHHAHDAYLNAVVGTA
LIKKYPKLESEFVYGDYKVYDVRKMIAKSEQEIGKATAKYFFYSNIMNFFKTEITLANGEIRKRPLIE
TNGETGEIVWDKGRDFATVRKVLSMPQVNIVKKTEVQTGGFSKESILPKRNSDKLIARKKDWDPK
KYGGFDSPTVAYSVLVVAKVEKGKSKKLKSVKELLGITIMERSSFEKNPIDFLEAKGYKEVKKDLII
KLPKYSLFELENGRKRMLASAGELQKGNELALPSKYVNFLYLASHYEKLKGSPEDNEQKQLFVEQ
HKHYLDEIIEQISEFSKRVILADANLDKVLSAYNKHRDKPIREQAENIIHLFTLTNLGAPAAFKYFDT
TIDRKRYTSTKEVLDATLIHQSITGLYETRIDLSQLGGD

NLS Cas9 variants

(color shown as magenta for HA tag, green for His_6 tag and yellow for NLS sequence)

N0/C0 SpyCas9

[SpyCas9 Core Sequence]-GGSYPYDVPDYALEGGGSHHHHHH

N0/C1 SpyCas9

[SpyCas9 Core Sequence]-GGSYPYDVPDYA
LEGGGRGGSPKKKRKVGGSHHHHHH

N0/C2 SpyCas9

[SpyCas9 Core Sequence]-GGSYPYDVPDYALEGGGSPKKKRKVGGRGGSPKKKRKVGGSHHHHHH

N0/C3 SpyCas9
[SpyCas9 Core Sequence]-
GGSYPYDVPDYALEGGGSPKKKRKVGGSPKKKRKVGGRGGSPKKKRKVGGSHHHHHH

N0/C4 SpyCas9
[SpyCas9 Core Sequence]-
GGSYPYDVPDYALEGGGSPKKKRKVGGSPKKKRKVGGSPKKKRKVGGRGGSPKKKRKVGGSHH
HHHH

N1/C0 SpyCas9
MGSSPKKKRKVGGSTS-**[SpyCas9 Core Sequence]**-GGSYPYDVPDYALEGGGSHHHHHH

N1/C1 SpyCas9(Yellow, NLS; magenta, HA; green, His_6 tag)
MGSSPKKKRKVGGSTS-**[SpyCas9 Core Sequence]-**
GGSYPYDVPDYALEGGGRGGSPKKKRKVGGSHHHHHH

N1/C2 SpyCas9
MGSSPKKKRKVGGSTS-**[SpyCas9 Core Sequence]-**
GGSYPYDVPDYALEGGGSPKKKRKVGGRGGSPKKKRKVGGSHHHHHH

N1/C3 SpyCas9
MGSSPKKKRKVGGSTS-**[SpyCas9 Core Sequence]-**
GGSYPYDVPDYALEGGGSPKKKRKVGGSPKKKRKVGGRGGSPKKKRKVGGSHHHHHH

N2/C0 SpyCas9
MGSSPKKKRKVGGSTSPKKKRKVGGS-**[SpyCas9 Core Sequence]-**
GGSYPYDVPDYALEGGGSHHHHHH

N2/C1 SpyCas9
MGSSPKKKRKVGGSTSPKKKRKVGGS-**[SpyCas9 Core Sequence]-**
GGSYPYDVPDYALEGGGRGGSPKKKRKVGGSHHHHHH

N2/C2 SpyCas9
MGSSPKKKRKVGGSTSPKKKRKVGGS-**[SpyCas9 Core Sequence]-**
GGSYPYDVPDYALEGGSPKKKRKVGGRGGSPKKKRKVGGSHHHHHH

N3/C0 SpyCas9
MGSSPKKKRKVGGSTSPKKKRKVGGSPKKKRKVGGS-**[SpyCas9 Core Sequence]-**
GGSYPYDVPDYALEGGGSHHHHHH

N3/C1 SpyCas9
MGSSPKKKRKVGGSTSPKKKRKVGGSPKKKRKVGGS-**[SpyCas9 Core Sequence]-**
GGSYPYDVPDYALEGGGRGGSPKKKRKVGGSHHHHHH

N4/C0 SpyCas9

MGSSPKKKRKVGGSTSPKKKRKVGGSPKKKRKVGGSPKKKRKVGGS-**[SpyCas9 Core Sequence]**-GGSYPYDVPDYALEGGGSHHHHHH
